# The Importance of Being First: Exploring Priority and Diversity Effects in a Grassland Field Experiment

**DOI:** 10.3389/fpls.2016.02008

**Published:** 2017-01-05

**Authors:** Emanuela W. A. Weidlich, Philipp von Gillhaussen, Benjamin M. Delory, Stephan Blossfeld, Hendrik Poorter, Vicky M. Temperton

**Affiliations:** ^1^Plant Sciences, Institute for Bio- and Geosciences-2, Forschungszentrum Jülich GmbHJülich, Germany; ^2^Ecosystem Functioning and Services, Institute of Ecology, Leuphana UniversityLüneburg, Germany

**Keywords:** plant functional group, biodiversity, assembly, order of arrival, historical contingency

## Abstract

Diversity of species and order of arrival can have strong effects on ecosystem functioning and community composition, but these two have rarely been explicitly combined in experimental setups. We measured the effects of both species diversity and order of arrival on ecosystem function and community composition in a grassland field experiment, thus combining biodiversity and assembly approaches. We studied the effect of order of arrival of three plant functional groups (PFGs: grasses, legumes, and non-leguminous forbs) and of sowing low and high diversity seed mixtures (9 or 21 species) on species composition and aboveground biomass. The experiment was set up in two different soil types. Differences in PFG order of arrival affected the biomass, the number of species and community composition. As expected, we found higher aboveground biomass when sowing legumes before the other PFGs, but this effect was not continuous over time. We did not find a positive effect of sown diversity on aboveground biomass (even if it influenced species richness as expected). No interaction were found between the two studied factors. We found that sowing legumes first may be a good method for increasing productivity whilst maintaining diversity of central European grasslands, although the potential for long-lasting effects needs further study. In addition, the mechanisms behind the non-continuous priority effects we found need to be further researched, taking weather and plant-soil feedbacks into account.

## Introduction

In ecology, the topics of biodiversity and ecosystem functioning (Balvanera et al., 2006; Isbell et al., 2011) and community assembly (Diamond, 1975; Fukami and Nakajima, 2011) are key components of the field, but have rarely being explicitly combined in experimental setups. Both the diversity of species as well as the order in which they arrive in the system can have strong effects on ecosystem functioning and community composition. As such, one might expect strong interactions between biodiversity and order of arrival. One key question is: to what extent would positive biodiversity effects found in biodiversity experiments sown at the same time be different if order of arrival was manipulated as well? Equally, within assembly experiments, what role does the diversity of the community play for establishment success? Biodiversity theory predicts that more diverse communities will be harder to invade (Elton, 1958), but evidence partly supports this theory (Hector et al., 2001; Fargione and Tilman, 2005) and partly does not (Stohlgren et al., 1999).

Since ecological communities are not static over time, understanding plant community assembly and how species can drive assembly has long been a primary goal for ecologists (Diamond, 1975; Connell and Slatyer, 1977). Within this context, the issue of historical contingency (dependence on history) is central, and involves the study of the effects of past events, whether biotic or abiotic (Drake, 1991; Eriksson and Eriksson, 1998; Fukami, 2015). This includes order of arrival of specific organisms as well as effects of disturbances (Drake, 1991; Temperton and Hobbs, 2004). The study of priority effects, in contrast, focuses solely on biotic effects, and happens when organisms that first arrive at a site can significantly affect the establishment, growth, or reproduction of the species arriving later, thus influencing further assembly (Eriksson and Eriksson, 1998; Fukami, 2015; Vaughn and Young, 2015). Priority effects can affect both the structure and functioning of ecosystems. In addition, priority effects can have a stronger influence on community composition than abiotic conditions (Fukami, 2015). As such, priority effects may be a powerful tool for ecological restoration, since the order of arrival or initial plant species composition can be manipulated in ecological restoration (Schantz et al., 2015; Vaughn and Young, 2015; Temperton et al., 2016). Priority effects, for example, may be useful for sending plant communities on desired trajectories for restoration.

Many experiments that test priority effects in plant communities are located in the United States, where the role of order of arrival of invasive exotic annual grasses (often from Europe) is often explored, since they can cause major species loss in native communities (Corbin and D’Antonio, 2004; Martin and Wilsey, 2012; Goldstein and Suding, 2014; Vaughn and Young, 2015). In contrast, for species-rich grasslands in Europe, the main threat for these habitats is not invasive species, but land use intensification as well as abandonment. Therefore, any incentive for farmers to keep extensively managing grasslands for diversity and higher productivity would be of benefit to species conservation in Europe (Bullock et al., 2007).

Biodiversity-ecosystem functioning (BEF) experiments have tested how species and functional richness affect ecosystem functioning in grasslands (Hector, 1999; Roscher et al., 2004), in aquatic (Callaway et al., 2003; Cardinale et al., 2009) and in forest systems (Bruelheide et al., 2014). Such experiments generally find positive diversity effects, with higher sown plant diversity leading to improved functioning of ecosystems, such as productivity, nutrient cycling. Often, positive effects found also increase over time. We know from BEF experiments in grasslands, that plant functional groups (PFGs) such as legumes, non-leguminous forbs, and grasses can positively affect ecosystems processes (Diaz and Cabido, 2001; Pokorny et al., 2005). Effects of species and functional group richness as well as different combinations of functional groups can produce positive diversity effects on ecosystem functions. Legumes combined with grasses often show particularly strong diversity effects (Oelmann et al., 2007; Temperton et al., 2007; Fornara and Tilman, 2008). Less is known about how relevant such BEF experiments are under natural assembly conditions (but see Bullock et al., 2007), since normally the species richness levels are maintained by weeding the plots. However, within the Jena Experiment (BEF) some studies have addressed assembly questions (e.g., Roscher et al., 2014) by stopping weeding and adding seeds and found that historical contingency was not eradicated by stopping to weed. When testing the relevance of positive biodiversity effects found in BEF experiments in a restoration context, Bullock et al. (2007) sowed different seed mixtures and then allowed communities to assemble naturally. They sowed either low or high diversity seed mixtures (sowing all species at the same time) on ex-arable land, and found that sowing species-rich mixtures only once positively affected both aboveground productivity and diversity over many years.

So far experiments manipulating plant species order of arrival have mainly used controlled experimental set-ups using pots or mesocosms (Ejrnæs et al., 2006; Chase, 2010; Moore and Franklin, 2011; Stevens and Fehmi, 2011; Dickson et al., 2012; Byun et al., 2013; Kardol et al., 2013; Mason et al., 2013; Ulrich and Perkins, 2014; Burkle and Belote, 2015; Wilsey et al., 2015; Sikes et al., 2016). Focusing on order of arrival of different PFGs, Körner et al. (2008) set up an experiment with nine grassland species from three different groups (non-leguminous forbs, legumes, and grasses), sowing one group before the other two. This stepwise arrival promoted different below and aboveground biomass depending on which functional group was sown first. They found priority effects of sowing legumes first, with more community biomass above and less belowground. In addition, von Gillhaussen et al. (2014) found that sowing legumes before the other functional groups affected assembly more than sowing density or sowing interval did.

With regard to field experiments, there are few studies testing order of arrival effect for more than one growing season: Collinge and Ray (2009) worked with vernal pools (wetlands), Fukami et al. (2005) manipulated initial colonization of native grasses on abandoned land, and Helsen et al. (2016) tested regeneration by removing specific functional groups from grasslands. Most of the studies testing priority effects by altering order of arrival compared effects of exotic and native competition both in the field (Chadwell and Engelhardt, 2008; Goldstein and Suding, 2014; Young et al., 2014; Vaughn and Young, 2015) and in controlled experiments (Grman and Suding, 2010; Stevens and Fehmi, 2011; Mason et al., 2013). These studies generally found that small differences in emergence timing can have long-lasting effects on community structure, and that initial control of exotics can increase the establishment of native perennial seedlings.

The strength of priority effects has been shown to differ depending on both soil nutrient content (Kardol et al., 2013), as well as on plant-soil feedback (Grman and Suding, 2010; van de Voorde et al., 2011; van der Putten et al., 2013). Kardol et al. (2013) found that effects of time of arrival depend on resource availability, and at high nutrient supply early arriving species grew quickly and reduced establishment of late arriving species.

Considering that diverse seed mixtures can improve diversity (Bullock et al., 2007), and that one can create priority effects by manipulating PFG order of arrival (Körner et al., 2008), we set up a field experiment combining these biodiversity and assembly approaches. We studied the effect of order of arrival of three PFGs (grasses, legumes, and non-leguminous forbs) and of sowing low and high diversity seed mixtures (9 or 21 species) on species composition and productivity on two different soil types. Our experiment is original since it combines biodiversity (sown diversity) and assembly (order of arrival) approaches, and moreover tests these factors on two different soil types. In general, we asked ourselves the question whether the effect of order of arrival is influenced by the sown diversity of the plant communities. At the same time, we wanted to know whether biodiversity effects as found in BEF experiments are influenced by order of arrival of PFGs, as the latter is usually not included as a factor in BEF experiments. Using our 4-year field experiment, we tested the following hypotheses:

(1) We expect PFG order of arrival to positively affect aboveground community biomass, with higher aboveground biomass in the treatments where legumes were sown first. We expect that PFG order of arrival will not affect the number of species but rather the functional composition of the community. More specifically, we expect that the PFG sown first will dominate each treatment (i.e., causing a priority effect).
(2) Sowing high diversity seed mixtures (sown diversity) will positively affect community aboveground biomass and number of species managing to establish.
(3) We expect an interaction effect between the order of arrival and sown diversity treatments. In particular, we hypothesize that the highest aboveground biomass will be found in the high diversity treatment where legumes were sown first.
(4) We expect that the outcome of PFG order of arrival and sown diversity will be modulated by soil type.

## Materials and Methods

### Experimental Site

The Priority Effect Experiment is located on an ex-arable field southeast of Jülich (Germany -altitude 94 m – 50°53′51.53″ N, 6°25′21.09″ E). Mean annual air temperature is 9.9°C and mean annual precipitation is 699 mm. The site was cultivated as an arable field until 2006 (mainly for the cultivation of vegetables and root crops) and was then used as extensive grassland (with typical grassland species sown by the farmer) from 2006 until the establishment of the experiment in 2012. Prior to the experiment the field was plowed and raked multiple times during the winter 2011/2012 to counteract germination of weeds from the soil seed-bank and to create bare ground.

**FIGURE 1:**
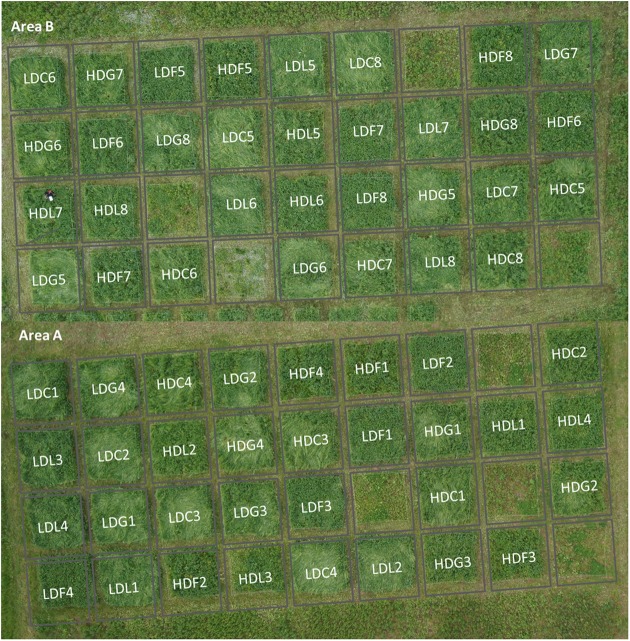
**Aerial image showing the experimental design used on the two sites (areas A and B).** Each plot is described by a code containing the following information: the sown diversity (HD, high diversity; LD, low diversity), the PFG order of arrival (F, forbs-first; G, grasses-first; L, legumes-first; and C, controls where all PFGs were sown at the same time) and the replicate number (*n* = 4 per area). The plots without any legend were the blank ones where nothing was sown (data not shown).

Four soil profiles were dug out in 2011 at the field site, and as result of it, the experiment was set up on two areas (A and B – **Figure 1**) reflecting the soil types Stagnic Cambisol (normally productive soil type) in area A and Anthrosol (modified by human activity) in area B (slightly elevated – approximately 1.8 m higher than A). The soil survey followed the official German soil mapping guidelines (Sponagel, 2005).

### Experimental Design and Species Selection

The main treatments of the experiment were the sown diversity (two levels: high or low diversity), the PFG order of arrival (four levels: grasses, forbs, or legumes sown first and all PFGs sown at the same time) and the soil type (two levels: areas A and B). Areas A and B had exactly the same treatment factors and four replicates (*n* = 4 per soil type) resulting in a total of 64 plots of 4 m × 4 m (**Figure 1**).

Two different sown diversity levels were used in the experiment to assess the effects of species richness on ecosystem functioning and diversity outcomes in the assembling communities. In total, a fixed set of 21 common species (seven forbs, seven grasses, and seven legumes) was selected for the high diversity communities. A randomly chosen, fixed subset of nine species (three forbs, three grasses, and three legumes) was selected to represent low diversity communities (**Table 1**). When choosing species, we aimed to reflect those which are relatively common and dominant in grasslands of the area. The target plant community is a semi-natural species-rich mesotrophic grassland, consisting of typical central European grassland species (Ellenberg, 1988). Species were selected taking their performance in previous controlled experiments (von Gillhaussen et al., 2014) and pre-experiments into account. Species were classified into three different PFGs: forbs (non-leguminous), grasses, and legumes. These species categories were intentionally kept broad, to create general functional envelopes which each include plant species that differ significantly in their functional and morphological traits (based on Roscher et al., 2004). Forbs included any non-leguminous, non-grass species. Grasses included members of the *Poaceae* family, and these species are morphologically most different from the other groups. Legumes are forbs of the *Fabaceae* family which differ from species of other PFGs in their ability to fix atmospheric N_2_.

**Table 1:** Plant species chosen for the Priority Effect Experiment with the respective PFG assigned for each species.

Plant functional groups (PFG)	Species	Code in PCA	Sown diversity:	
			
			High	Low
Forbs	*Achillea millefolium*	F1	x	x
	*Crepis biennis*	F2	x	
	*Galium verum*	F3	x	
	*Geranium pratense*	F4	x	
	*Leontodon hispidus*	F5	x	
	*Leucanthemum vulgare*	F6	x	x
	*Plantago lanceolata*	F7	x	x
Grasses	*Arrhenatherum elatius*	G1	x	
	*Bromus erectus*	G2	x	
	*Dactylis glomerata*	G3	x	x
	*Festuca pratensis*	G4	x	x
	*Helictotrichon pratense*	G5	x	
	*Holcus lanatus*	G6	x	x
	*Poa pratensis*	G7	x	
Legumes	*Lathyrus pratensis*	L1	x	
	*Lotus corniculatus*	L2	x	x
	*Medicago sativa*	L3	x	x
	*Onobrychis viciifolia*	L4	x	
	*Trifolium hybridum*	L5	x	
	*Trifolium pratense*	L6	x	x
	*Trifolium repens*	L7	x	


*Species were selected from a species pool of the typical central European grassland types. Species pools for high and low diversity (HD and LD) mixtures were fixed (not random). Presence of species in a sown diversity is denoted by an “x.”*

The PFG order of arrival treatment was created by sowing the species of one PFG on April 19th, 2012 (or all PFGs at the same time in the control plots), while the species from the other PFGs were sown on May 31st, 2012, resulting in four treatment levels: forbs-first (F-first), grasses-first (G-first), legumes-first (L-first), and control. The length of the interval between sowing events was based on a previous greenhouse study (von Gillhaussen et al., 2014), where a 6-week interval produced larger priority effects than a 3-week interval. Before the 2nd sowing all plots were mown, to allow subsequently sown species a better chance to germinate and establish, and to increase complementarity between PFGs. None of the plots was weeded thus allowing colonization and natural assembly processes to occur after the sowing events.

In each plot, the sowing density was 5 g/m^2^ divided equally among the species of each mixture. The seed mixtures were mixed with sand to improve handling and ensure an even distribution on the plots at the time of sowing. The number of seeds taken for each species was adjusted according to their thousand seed weight. Seeds were sown by hand into the plots, and afterward each plot was flattened to ensure proper adherence of seeds to soil particles and to avoid granivory. A non-clonal grass species, *Festuca rubra spp. commutatis*, was sown in the areas between the plots as lawn paths.

### Sampling and Data Collection

To assess the effects of our treatments on community composition, we estimated the plant cover of each species prior to the harvest of aboveground biomass using the *Braun-Blanquet* method modified by Londo (1976). We assessed the cover of non-target (mainly weedy) species as a total cover for this group, but this data is not shown in the graphs. Since the non-target species were not identified down to species level and hence could not be assigned to different PFGs, we decided to exclude them from the analyses. Even though the weed cover in the 1st year was up to 20%, this reduced drastically due to mowing over the years (<1% in 2015).

Total aboveground biomass (dry matter yield, g/m^2^) was measured in June (2012, 2013, 2014, and 2015) and September (2012, 2013, and 2014). Here, we only report the peak biomass data from June of each year. Two randomly positioned 0.1 m^2^ rectangles (20 × 50 cm) were harvested from each plot at each harvest. All aboveground plant material within the rectangle was cut 2 cm above the soil surface and samples were dried at 70°C (until constant weight) before weighing. During the harvest of 2014, harvested plant material was sorted into species, in order to have biomass data per species. All plots were mown twice per growing season (according to agricultural practice in managed mesotrophic grasslands), in July and September, except in 2015 when we harvested only once at peak biomass in June.

Total carbon (C), nitrogen (N), potassium (K), and phosphorus (P) in soil samples were measured in April, 2012 and in September, 2014 by pooling three soil cores (each 40 cm × 5 cm) into one sample per plot, giving a total of 64 soil samples per element. Soil samples collected from each plot of the experiment were analyzed for %C, %N, %K, and %P (VarioelCube Elementar and ICP-OES methods). For %P in soil, we were only able to analyse the samples from 2014, since in 2012 the measurements were below the detection limit of the method.

### Statistical Analyses

Our field experiment was performed over 4 years and is multi-factorial in design, with PFG order of arrival and sown diversity as the main fixed factors. Because, we were interested to see if the effects of PFG order of arrival and sown diversity on the measured variables changed between experimental sites, we also considered soil type as a fixed factor. All statistical analyses were performed using R 3.3.1 (R Core Team, 2016) and an alpha value of 5%.

The effects of treatments on aboveground biomass and on species richness were analyzed with linear mixed effects models following the procedure described by Zuur et al. (2009). For each variable, we started by fitting a model containing all explanatory variables and all possible interactions between PFG order of arrival, sown diversity and soil type. First, we found the optimal structure of the random component of each model using restricted maximum likelihood (REML) estimators. Using year as a random factor, we compared two different random structures: (1) no random term (using the generalized least squares method) and (2) a random intercept model. The model with the lowest Akaike Information Criterion (AIC) value was then selected. For both aboveground biomass and species richness, a random intercept model was retained for further statistical analyses. We then found the optimal fixed structure of each model by dropping the non-significant terms (based on *F*-statistics). The linear mixed models were fitted with the lme function of the nlme package (Pinheiro et al., 2016).

Because, we were interested to see if each of the factors tested in our grassland experiment had an effect on aboveground biomass and species richness on each soil type in each year, the data were also analyzed using one-way ANOVAs, where either PFG order of arrival or sown diversity were the fixed factor. When the null hypothesis was rejected, the mean treatment values were compared with a Newman–Keuls test performed with the R package agricolae (de Mendiburu, 2015).

The influence of PFG order of arrival and sown diversity on species composition over the years was analyzed using a principal component analysis (PCA). A single PCA was performed on a dataset containing the cover data of 21 plant species (2012, 2013, and 2015) using the R package FactoMineR (Husson et al., 2016). The PCA was performed using a correlation matrix constructed from scaled variables. In this paper, we considered that a variable (plant species) contributed significantly to a principal component (PC) if its contribution (expressed in %) was greater than the contribution that would have been observed if all variables contributed equally to a component. In our case, this threshold value was equal to 4.8% and was calculated as 100 divided by the number of species for which cover data were available (21).

We analyzed the soil chemistry data using linear models, because we were interested to see the effects of our experimental factors (PFG order of arrival, sown diversity) as well as soil type and year. For each of the four soil variables (C, N, P, and K), we started by fitting a model containing all explanatory variables and all possible interactions between the factors. Then, we simplified the model by dropping the non-significant terms based on *F*-statistics. The linear models were fitted using the lm function of R.

## Results

### Effects of PFG Order of Arrival and Sown Diversity on Aboveground Biomass

**FIGURE 2:**
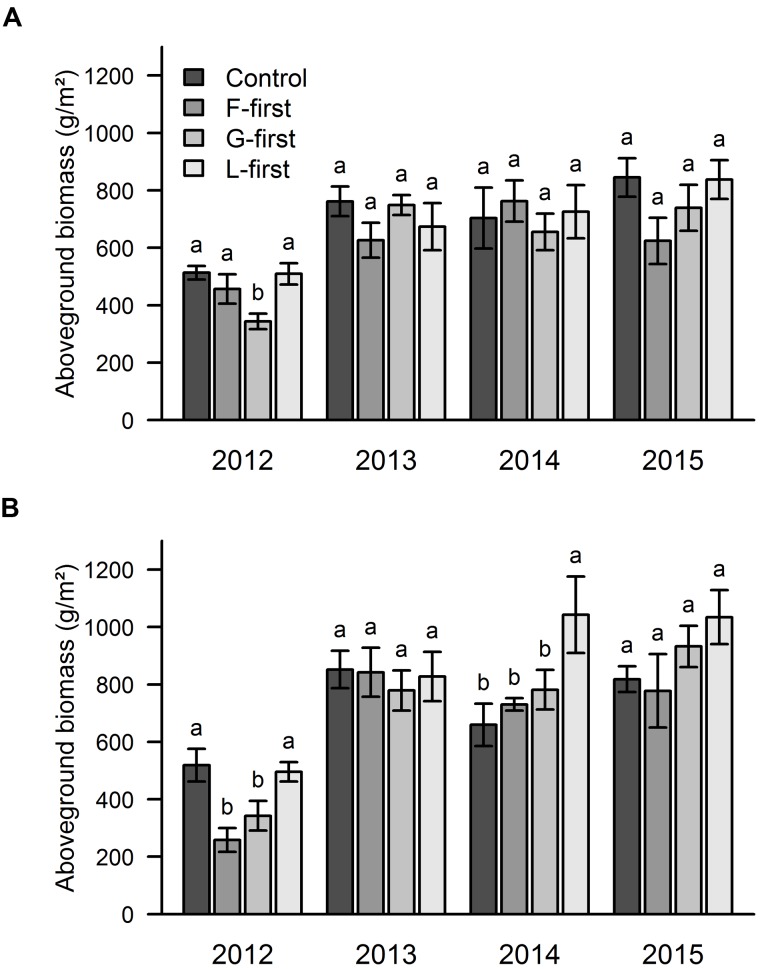
**Influence of PFG order of arrival on aboveground productivity over 4 years of a grassland experiment.** The results are shown separately for the two experimental sites: area A **(A)** and area B **(B)**. In control plots, all PFGs were sown at the same time. In the other plots, the PFG order of arrival was experimentally manipulated (F-first, forbs sown first; G-first, grasses sown first; L-first, legumes sown first). The values are means ± one standard error of the mean (*n* = 4). Within each year, different letters show significant differences between treatments (one-way ANOVA followed by a Newman–Keuls test, *P* < 0.05).

**FIGURE 3:**
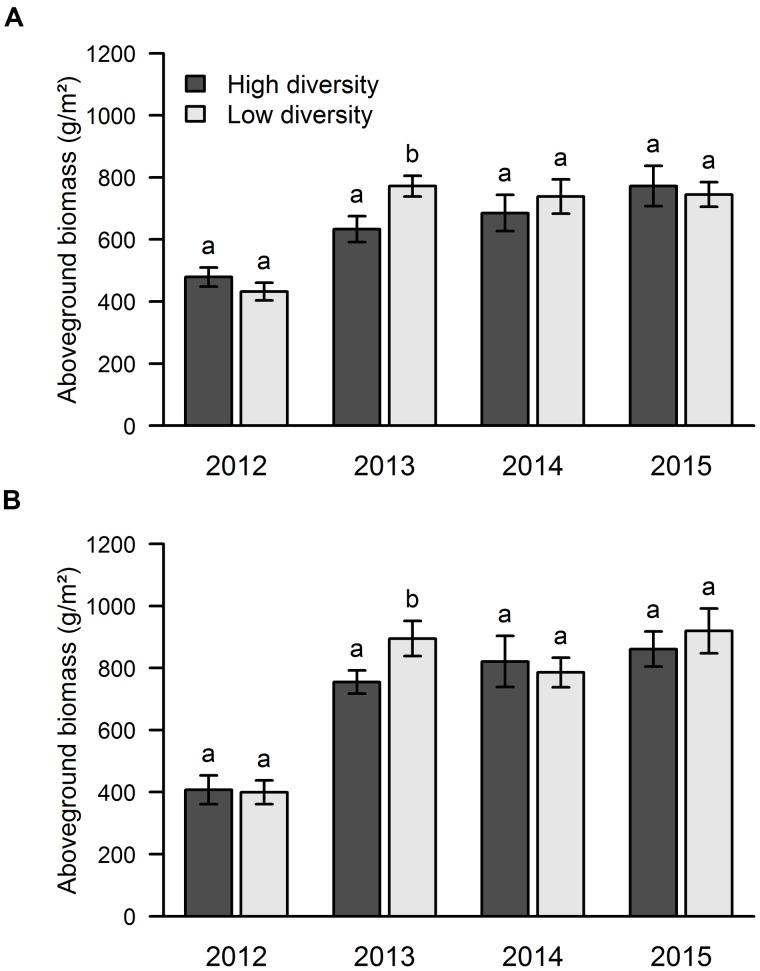
**Influence of sown diversity on aboveground productivity over 4 years of a grassland experiment.** The results are shown separately for the two experimental sites: area A **(A)** and area B **(B)**. A total of 21 and 9 species were sown at the beginning of the experiment in high and low diversity plots, respectively. The values are means ± one standard error of the mean (*n* = 4). Within each year, different letters show significant differences between treatments (one-way ANOVA followed by a Newman–Keuls test, *P* < 0.05).

The mixed effect model run over the whole 4-year dataset showed that the aboveground biomass was significantly affected by PFG order of arrival (*P* = 0.0011), but not by sown diversity. We did not find any significant interaction between PFG order of arrival, sown diversity and soil type, therefore, the graphs are shown separately for each factor (without interation). Since, we were explicitly interested in how sown diversity and order of arrival affected the biomass over time, we also analyzed the effects of PFG order arrival (**Figure 2**) and sown diversity (**Figure 3**) on aboveground biomass separately for each year and each soil type. The mean biomass over the 4 years was 656 g/m^2^ in area A and 731 g/m^2^ in area B.

Our results from 2012 showed that the highest biomass values were obtained when all PFGs were sown at the same time, and when forbs (area A) or legumes (areas A and B) were sown first (**Figure 2**). In the 2nd year of the experiment (2013), there were no significant differences in biomass between order of arrival treaments (**Figure 2**). In 2014, we found that legumes promoted priority effects only in area B, where the legumes-first treatment had significantly higher biomass than the other treatments (**Figure 2**). The same pattern was found in 2015, but this time without any statistically significant differences (**Figure 2**).

Looking at the effect of sown diversity, we found that the low diversity plots had higher biomass in 2013, while no significant differences were found in the other years (**Figure 3**).

### Effects of PFG Order of Arrival and Sown Diversity on Number of Species

The results of the mixed effects model showed that the number of species that managed to establish themselves was affected by PFG order of arrival (*P* = 0.0004) and sown diversity (*P* < 0.0001). We also found a significant interaction between sown diversity and soil type (*P* = 0.015). No other significant interaction were observed. The mean number of species in area A was slightly higher than in area B (7.7 and 6.5 species per plot, respectively).

**FIGURE 4:**
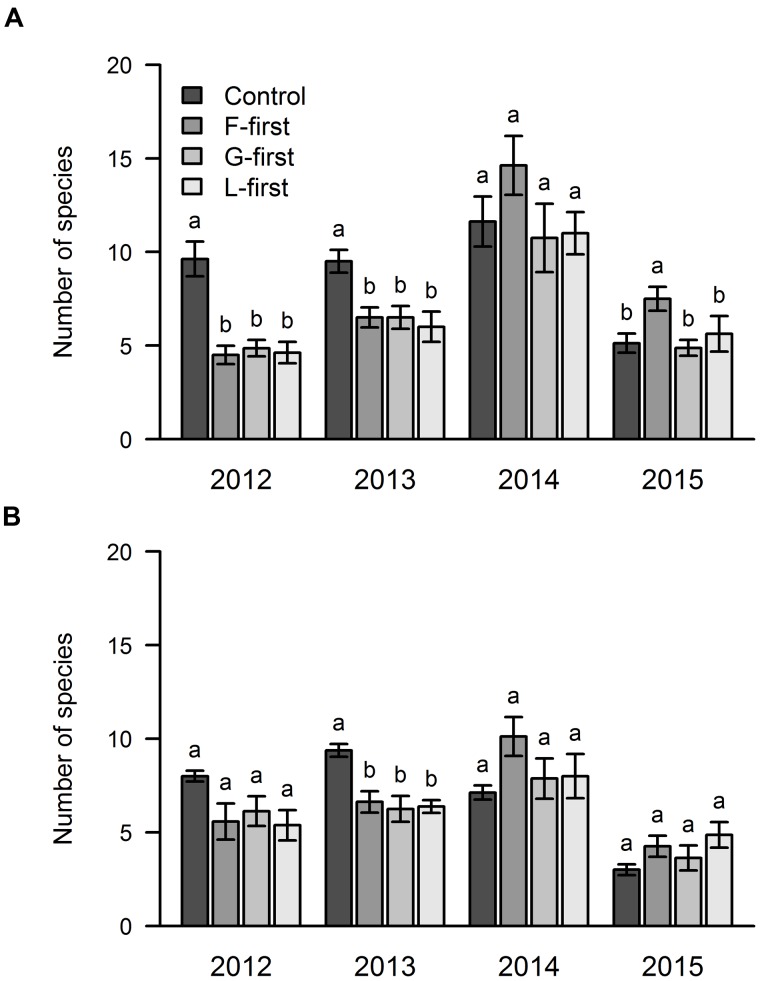
**Influence of PFG order of arrival on species richness over 4 years of a grassland experiment.** The results are shown separately for the two experimental sites: area A **(A)** and area B **(B)**. In control plots, all PFG were sown at the same time. In the other plots, the PFG order of arrival was experimentally manipulated (F-first, forbs sown first; G-first, grasses sown first; L-first, legumes sown first). The values are means ± one standard error of the mean (*n* = 4). Within each year, different letters show significant differences between treatments (one-way ANOVA followed by a Newman–Keuls test, *P* < 0.05).

Our results showed that PFG order of arrival significantly affected the number of species in the 1st (area A) and 2nd year (areas A and B) of the experiment because the control treatment had a higher number of species in comparison with the other treatments (**Figure 4**). In 2014, we did not find any statistical difference between the treatments in both areas with regard to the species richness. In the following year (2015), we found significantly more species in the F-first plots of area A in comparison with the other plots of the same area, but in area B, the species richness was not affected by PFG order of arrival.

**FIGURE 5:**
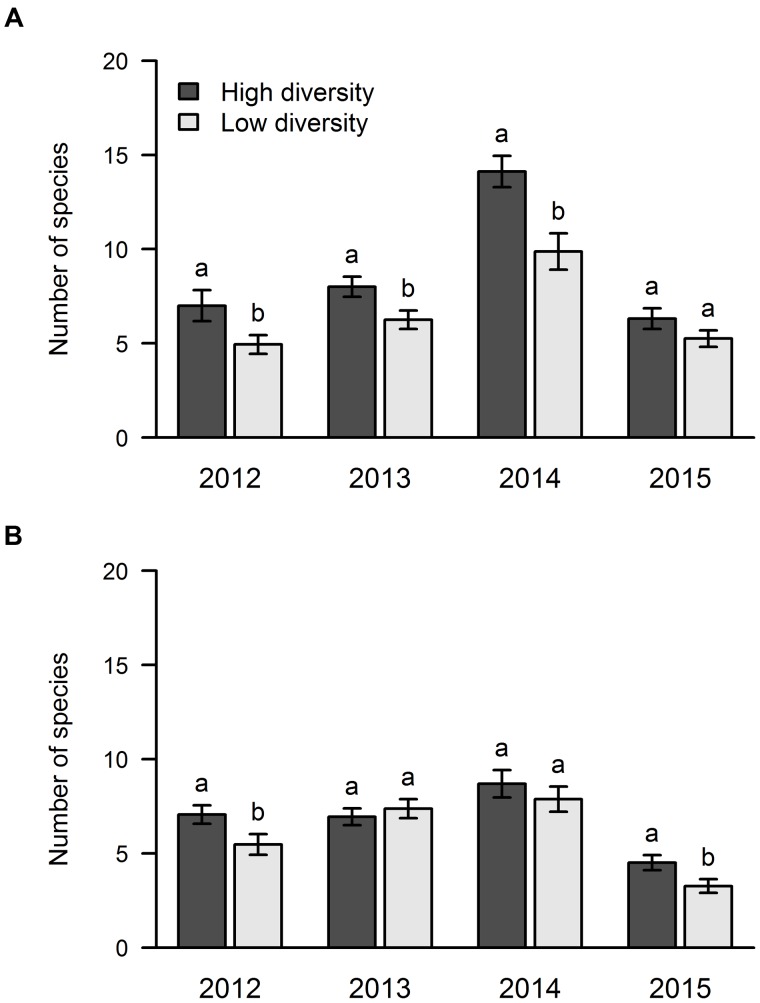
**Influence of sown diversity on species richness over 4 years of a grassland experiment.** The results are shown separately for the two experimental sites: area A **(A)** and area B **(B)**. A total of 21 and 9 species were sown at the beginning of the experiment in high and low diversity plots, respectively. The values are means ± one standard error of the mean (*n* = 4). Within each year, different letters show significant differences between treatments (one-way ANOVA followed by a Newman–Keuls test, *P* < 0.05).

With regard to the effect of sown diversity, the number of species that managed to establish was higher in the high diversity treatment in areas A and B in 2012, while only area A showed this pattern in 2013 and 2014 (**Figure 5**). In 2015, we found more species in the high diversity plots only in area B (**Figure 5**).

### Effects of PFG Order of Arrival and Sown Diversity on Community Composition

The PCA showed that in the 1st (2012) and 2nd (2013) year of the experiment, the F-first, G-first, and L-first plots mainly consisted of the plant species belonging to the PFG sown first, as expected. In 2012, the species composition of the control plots differed between the two sown diversity levels. When the control plots were sown in low diversity sowing (positive PC2 values), they had on average a greater forb cover than the control plots sown in the high diversity sowing, which were composed mainly by legumes (negative PC2 values). In contrast to the first 2 years, however, from 2013 to 2015, the species composition of the plots in all treatments converged to a state dominated by two grasses (*Holcus lanatus* and *Dactylis glomerata*) regardless of the PFG order of arrival and sown diversity treatments.

**FIGURE 6:**
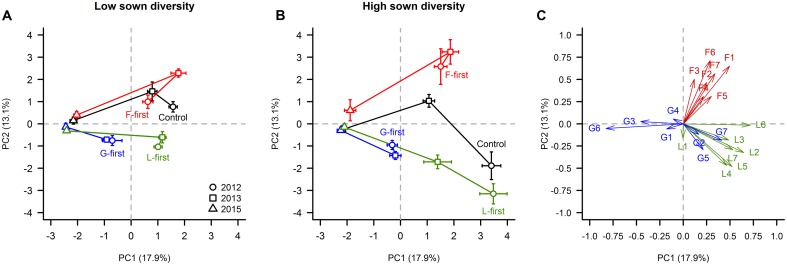
**Influence of PFG order of arrival on species composition.** Cover data were analyzed with a PCA. In panels **(A)** and **(B)**, a score plot constructed with the two first principal components (PC) is shown for each sown diversity level. The panel **(C)** shows the correlation coefficient of each plant species with PC1 and PC2. Horizontal and vertical error bars are standard errors according to PC1 and PC2, respectively. In panel **(C)**, we used a code made of one letter (refers to the PFG; F = forbs, G = grasses, L = legumes) and one number for naming the plant species. A full description of the code can be found in **Table 1**.

More specifically, the two first PCs accounted for 31% of the total variance. The species *H. lanatus* (18.1%), *Trifolium pratense* (13.6%), *Lotus corniculatus* (11.1%), *T. repens* (7.5%), *T. hybridum* (7.2%), *Achillea millefolium* (6.5%), *Medicago sativa* (6.3%), *Onobrychis viciifolia* (5.7%), and *D. glomerata* (5.5%) contributed significantly to PC1 (**Figures 6A,B**). Essentially, PC1 can be interpreted as an axis separating plots dominated by *H. lanatus* and *D. glomerata* from plots dominated by forbs and legumes (**Figure 6**). With regard to PC2, the plant species that had the greatest contributions were *L. vulgare* (18.4%), *A. millefolium* (15.4%), *Plantago lanceolata* (11.6%), *Galium verum* (9.2%), *T. hybridum* (8.4%), *O. viciifolia* (8.0%), and *Crepis biennis* (7.9%), and mainly separated plots dominated by forbs from the ones dominated by legumes.

### Soil Chemistry

Our experimental factors (sown diversity and PFG order of arrival) had no significant effect on the soil parameters analyzed in both years. Data analyses for soil C, N, and K showed a highly significant interaction between year and soil type (C: *P* < 0.0001; N: *P* < 0.0001; K: *P* = 0.0009).

Soil samples from area B contained significantly more C and N in 2014, whereas in 2012, these parameters did not differ between the two experimental sites. Soil K in area A was higher than in area B only in 2012, while the measured values did not differ between areas in 2014. We observed strong decrease of soil K from 2012 to 2014 (**Supplementary Table 1**).

## Discussion

We found a transient significant effect of PFG order of arrival over the 4 years on aboveground biomass, number of species, and species composition. We could partially confirm our first hypothesis that PFG order of arrival would affect biomass and species composition, but could not confirm an effect on the number of species. The effects of order of arrival found on biomass and species composition were only partially confirmed since they changed over time. In the 1st year, aboveground biomass was significantly higher in L-first and in control plots than in G-first and F-first plots. This effect was probably related to these treatments having similar species compositions. In the 2nd year this pattern disappeared, while in the 3rd year, we found significantly more aboveground community biomass in legume-first plots, but only in area B. Our results suggest that legume order of arrival was partly driving aboveground biomass, but that this legume effect was not continuous over time, such that we did not find a clear continuous priority effect. Our field experiment results are nevertheless consistent with outcomes in similar controlled experiments (Körner et al., 2008; von Gillhaussen et al., 2014), in that legumes played an important role in our field experiment, albeit not as clearly as found under controlled conditions.

The two main processes behind priority effects are niche pre-emption and niche modification (Fukami, 2015). In the first mechanism, species that arrive first have the advantage of early establishment, and hence tend to perform better due to asymmetric competition. In the second mechanism, early-arriving species directly modify the abiotic environment, thereby changing the type of niches that are available, thus affecting establishment opportunities for later arriving species that colonize the community. Legumes grow fast aboveground and forbs generally invest proportionally less in root biomass than grasses as seen in Körner et al. (2008) and Poorter et al. (2015), and found in 2012 and 2014 in our experiment (data not shown here). A possible mechanism for the sporadic legume priority effect we found in the field, is that the PFG arriving after legumes had more opportunities for root and nutrient foraging due to the smaller root systems of the legumes that arrived first (a process known as N sparing; Felten et al., 2016). Since (1) the species composition of control and legumes-first plots was very similar in the 1st year (dominated by legumes) and (2) we found a significant legumes-first priority effect in the 3rd year, probably the N sparing mechanism could have played a role in our system. For some reason, however, this effect was not consistent over time. It is also possible that later neighbors of legumes benefitted from N transfer, where the neighbors of N_2_-fixers profit from legume-fixed N via direct or indirect transfer (*sensu* Temperton et al., 2007; Bessler et al., 2012).

The number of species found in plots in 2012 and 2013 show strong priority effects of sowing a PFG before another, since the number of species establishing was higher when all PFGs were sown at the same time. However, in the 3rd year the species richness was no longer different between the treatments, while in 2015 more species were found in the F-first plots (although only in area A). These results partially agree with our first hypothesis, that PFG order of arrival would not affect the number of species as much as the species composition. With regard to the latter, we expected that the PFG sown first would dominate each treatment, possibly causing a priority effect, and our results showed this in the 1st and 2nd year. The F-first, G-first, and L-first plots were mainly covered by the plant species belonging to the PFG sown first, while in control plots, the legumes were characterized by a slightly higher cover than the average. On the other hand, the following years showed that the plots had higher cover of grasses (mainly *H. lanatus* and *D. glomerata*). It seems that the community composition was possibly tracking natural grassland succession in the experiment, where legumes are often more common in the beginning, with grasses gradually taking over.

Our results agree with Ejrnæs et al. (2006) who found that species richness and invasibility is controlled by environment but that species composition is determined by plant order of arrival. This emphasizes that historical contingency can significantly change the outcome of community assembly. In contrast, another study concluded that historical effects may be lost within a decade, and found significant but transient effects of seeding, order of colonization, and frequency of colonization on species abundance (Collinge and Ray, 2009). Experiments testing a 2-week planting advantage between exotic and native plant species (in field conditions) also found that although priority effects reduced over time, small differences in emergence timing had long-lasting effects in the community (Vaughn and Young, 2015).

Our second hypothesis that sown diversity would promote higher aboveground biomass and number of species was confirmed for the effect on number of species but not for biomass. As expected, sown diversity clearly affected the number of species that established. The high diversity treatments usually had a higher number of species but we did not find a significant effect of sown diversity on biomass. This is in contrast to most of the BEF experiments, which normally find an effect of sown diversity on aboveground biomass and other ecosystem processes (Balvanera et al., 2006; Marquard et al., 2009).

The lack of significant effects of sowing high or low diversity seed mixtures on biomass in 2012, 2014, and 2015 might be explained by the fact that our low diversity treatment consisted of rather dominant species within the three PFGs. Thus, these dominant species could also perform very well in the low diversity treatment, and were more competitive than the additional species in the high diversity (21 species) treatment. In the Bullock et al. (2001, 2007) studies that found higher aboveground biomass when sowing more diverse seed mixtures (as we expected to find), most of the low diversity treatments did not include legume species, while all the high diversity treatments did. This may explain why they found more sustained effects of sown diversity over time than was found in our study (which had legumes in both treatments).

In our third hypothesis, we expected that L-first plots sown with high diversity seed mixtures would have the highest biomass. But since there was no significant interaction between PFG order of arrival and sown diversity, this hypothesis was not confirmed by our results. As stated above, it is possible that the species present in the low diversity plots tended to be quite dominant over time, thus hindering the effect of sown diversity and its interaction with order of arrival. On the other hand, we know from other *BEF* experiments that the species driving positive biodiversity effects are often the dominant ones, the identities of which change each year (Allan et al., 2011).

The lack of experiments combining biodiversity and assembly effects (sown diversity and PFG order of arrival) emphasizes the need for more studies to test this interaction over a range of different soil types and habitats, as well as taking species dominance explicitly into account.

Since we found a significant interaction between soil type and sown diversity, but did not find such interaction between soil type and PFG order of arrival, we could only partially confirm our fourth hypothesis that differences in soil type would affect the outcome of priority effects. Overall, irrespective of experimental treatments, area A with less fertile soil had a lower biomass but a higher number of species, while area B with more fertile soil had a higher biomass and lower number of species. Soil K decreased significantly between 2012 and 2014 but this was not affected by the experimental factors. Since K is essential for N_2_-fixation in legumes (Pda-Potash Development Association, 2015), this may have partly driven the overall change in dominance from legumes to grasses (irrespective of experimental treatments). This outcome of our experiment is thus not a desirable outcome for grassland restoration, where the goal is to develop a predominance of forbs mixed in with grasses, as is commonly found in central European grasslands (Kirmer et al., 2012). We now therefore suggest further research into the reason and effects of the reduction in soil K. Even though, we could not find significant interactions between soil type and PFG order of arrival, priority effects were more evident in area B. Since area B had higher concentrations of soil C and N than area A in 2014, plants in this area probably had enough nutrients and invested more in shoot growth than in roots thus promoting higher aboveground biomass. Similar results showing that plant species establishment depends on resource availability were found in a mesocosm experiment, where at high nutrient supply early arriving species grew quickly and reduced establishment of late arriving species (Kardol et al., 2013).

Ideally, to be able to use biodiversity and order of arrival effects as tools for economically viable ecological restoration in cultural landscapes, the legumes-first treatment would lead to higher community biomass at least in some years. In addition, functional group composition would ideally be more balanced than in the other treatment levels, but species number would not be affected by it (based on the greenhouse experiment of von Gillhaussen et al., 2014). Even though, we only partly found clear priority effects in our experiment, the results may have implications for restoration. For instance, if the non-continuous priority effects we found were more general in nature, this would mean that using such priority effects to steer communities along a desired trajectory would not be a viable procedure for restoration management. Thus, further experiments are now needed to be able to clarify whether manipulating order of arrival may be useful in a restoration context or not, using a range of different soil types and grassland habitats.

Our research suggests that sowing legumes first may be a valuable tool in creating more productive yet diverse communities in central European grasslands, but this may be context-dependent. The potential for long-lasting effects needs further study in different soil types and with different grassland types (e.g., oligotrophic vs. mesotrophic). In addition, understanding the mechanisms behind the non-continuous priority effects we found needs to be tested using a range of different experiments that address issues of weather and plant soil feedback (van de Voorde et al., 2011).

## Author Contributions

VT and PG designed and established the experiment. EW, PG, and VT collected the field data. EW, PG, and BD analyzed the data. EW and VT led the writing. HP and SB reviewed the manuscript. VT coordinated the study.

## Conflict of Interest Statement

The authors declare that the research was conducted in the absence of any commercial or financial relationships that could be construed as a potential conflict of interest.
