# Supplementary material for: The Importance of Being First: Exploring Priority and Diversity Effects in a Grassland Field Experiment
Source: Front Plant Sci. 2017 Jan 5;7:2008. doi: 10.3389/fpls.2016.02008 (PMC5221677; doi:10.3389/fpls.2016.02008)
Supplement: Supplementary file 1 [file Table_1.docx]

Supplementary Material

**THE IMPORTANCE OF BEING FIRST: EXPLORING PRIORITY EFFECTS OVER TIME IN A GRASSLAND FIELD EXPERIMENT**

**Emanuela W.A. Weidlich^1,2*^, Philipp von Gillhaussen^2,3^, Benjamin M. Delory¹, Stephan Blossfeld^2^, Hendrik Poorter^2^, Vicky M. Temperton^1,2^**

*** Correspondence:** Corresponding Author. [emanuela.weidlich@leuphana.de](mailto:emanuela.weidlich@leuphana.de)

# Supplementary Table

**
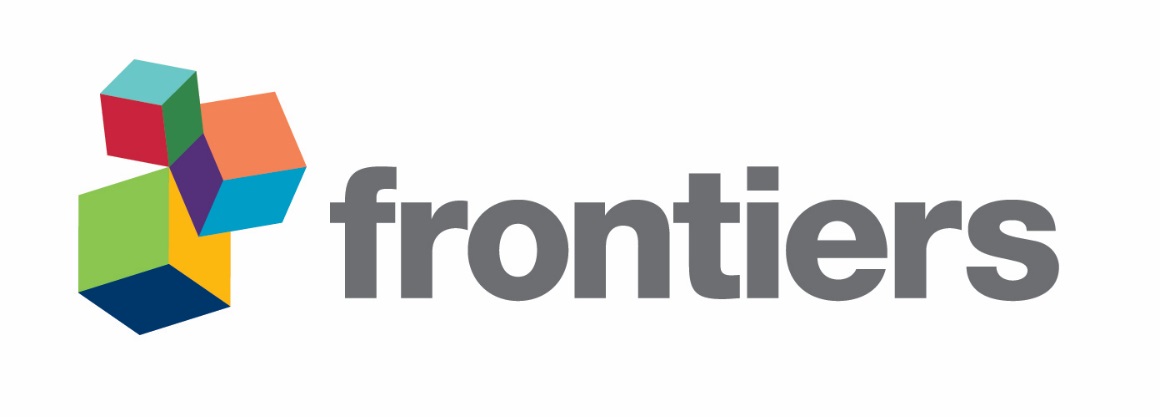
**

**Supplementary Table.** Mean percentage of soil nitrogen (N), phosphorus (P), potassium (K) and carbon (C) in the sown diversity and PFG order of arrival treatments, separated by year (2012, 2014) and areas A and B. Standard deviation is indicated by ±.

| **Sown diversity** | **PFG order of arrival** | **Soil chemistry** | | | | | | | | | | | | | |  |
| --- | --- | --- | --- | --- | --- | --- | --- | --- | --- | --- | --- | --- | --- | --- | --- | --- |
|  |  | **2012** | | | | | |  | **2014** | | | | | | |  |
|  |  | **% C** | | **% N** | | **% K** | |  | **% C** | | **% N** | | **% K** | | **% P** | |
| **AREA A** | | | | | | | | | | | | | | | | |
| High diversity | Control | 0.969 | ± 0.02 | 0.094 | ± 0.04 | 1.633 | ± 0.00 |  | 1.059 | ± 0.09 | 0.106 | ± 0.01 | 0.067 | ± 0.01 | 1.489 | ± 0.06 |
|  | F-first | 0.931 | ± 0.05 | 0.093 | ± 0.05 | 1.578 | ± 0.00 |  | 1.004 | ± 0.17 | 0.102 | ± 0.01 | 0.068 | ± 0.00 | 1.573 | ± 0.04 |
|  | G-first | 0.935 | ± 0.04 | 0.093 | ± 0.04 | 1.598 | ± 0.00 |  | 0.898 | ± 0.05 | 0.092 | ± 0.00 | 0.084 | ± 0.00 | 1.555 | ± 0.08 |
|  | L-first | 0.979 | ± 0.06 | 0.096 | ± 0.02 | 1.665 | ± 0.00 |  | 0.983 | ± 0.11 | 0.105 | ± 0.01 | 0.070 | ± 0.01 | 1.633 | ± 0.01 |
|  |  |  |  |  |  |  |  |  |  |  |  |  |  |  |  |  |
| Low diversity | Control | 0.966 | ± 0.04 | 0.094 | ± 0.06 | 1.595 | ± 0.00 |  | 1.030 | ± 0.03 | 0.109 | ± 0.00 | 0.069 | ± 0.02 | 1.518 | ± 0.04 |
|  | F-first | 0.961 | ± 0.06 | 0.094 | ± 0.07 | 1.528 | ± 0.00 |  | 1.015 | ± 0.06 | 0.100 | ± 0.00 | 0.088 | ± 0.01 | 1.623 | ± 0.05 |
|  | G-first | 0.972 | ± 0.03 | 0.095 | ± 0.06 | 1.690 | ± 0.06 |  | 0.953 | ± 0.10 | 0.097 | ± 0.01 | 0.079 | ± 0.01 | 1.543 | ± 0.09 |
|  | L-first | 0.971 | ± 0.02 | 0.096 | ± 0.02 | 1.645 | ± 0.00 |  | 0.949 | ± 0.19 | 0.107 | ± 0.01 | 0.072 | ± 0.01 | 1.595 | ± 0.05 |
| **AREA B** | | | | | | | | | | | | | | | | |
| High diversity | Control | 0.949 | ± 0.03 | 0.098 | ± 0.15 | 1.568 | ± 0.00 |  | 1.218 | ± 0.08 | 0.128 | ± 0.01 | 0.079 | ± 0.00 | 1.548 | ± 0.04 |
|  | F-first | 0.964 | ± 0.04 | 0.098 | ± 0.09 | 1.575 | ± 0.01 |  | 1.114 | ± 0.07 | 0.122 | ± 0.01 | 0.071 | ± 0.00 | 1.550 | ± 0.04 |
|  | G-first | 1.022 | ± 0.07 | 0.102 | ± 0.08 | 1.608 | ± 0.00 |  | 1.064 | ± 0.12 | 0.115 | ± 0.01 | 0.067 | ± 0.02 | 1.485 | ± 0.15 |
|  | L-first | 0.960 | ± 0.09 | 0.098 | ± 0.09 | 1.548 | ± 0.00 |  | 1.226 | ± 0.12 | 0.133 | ± 0.01 | 0.076 | ± 0.01 | 1.569 | ± 0.08 |
|  |  |  |  |  |  |  |  |  |  |  |  |  |  |  |  |  |
| Low diversity | Control | 0.950 | ± 0.02 | 0.099 | ± 0.04 | 1.580 | ± 0.00 |  | 1.169 | ± 0.08 | 0.135 | ± 0.01 | 0.068 | ± 0.02 | 1.575 | ± 0.04 |
|  | F-first | 0.961 | ± 0.04 | 0.095 | ± 0.16 | 1.485 | ± 0.00 |  | 1.214 | ± 0.21 | 0.133 | ± 0.02 | 0.074 | ± 0.01 | 1.615 | ± 0.04 |
|  | G-first | 0.971 | ± 0.06 | 0.100 | ± 0.08 | 1.480 | ± 0.01 |  | 1.152 | ± 0.08 | 0.126 | ± 0.01 | 0.080 | ± 0.00 | 1.625 | ± 0.05 |
|  | L-first | 0.931 | ± 0.05 | 0.095 | ± 0.07 | 1.465 | ± 0.00 |  | 1.141 | ± 0.04 | 0.128 | ± 0.00 | 0.075 | ± 0.01 | 1.600 | ± 0.00 |
